# The circulating renin-angiotensin-aldosterone system is down-regulated in dogs with glomerular diseases compared to other chronic kidney diseases with low-grade proteinuria

**DOI:** 10.1371/journal.pone.0262121

**Published:** 2022-01-10

**Authors:** Lisa-Maria Grandt, Ariane Schweighauser, Alan Kovacevic, Thierry Francey

**Affiliations:** Small Animal Internal Medicine, Vetsuisse Faculty University of Bern, Bern, Switzerland; Max Delbruck Centrum fur Molekulare Medizin Berlin Buch, GERMANY

## Abstract

Glomerular diseases (GD) lead to a variety of disorders of the vascular and the total body water volumes. Various pathomechanisms, including vascular underfill and overfill, have been suggested to explain these disturbances. Accordingly, the circulating renin-angiotensin-aldosterone system (cRAAS) is expected to be activated as either a cause or a result of these fluid disorders. The aim of this study was to characterize the activity of the cRAAS in dogs with GD and to evaluate its relationship with the vascular volume status. In a prospective study, we evaluated the plasma renin activity and the serum aldosterone concentration in 15 dogs with GD. Their fluid volume status was estimated with clinical variables reflecting volemia and hydration, echocardiographic volume assessment, N-terminal pro B-type natriuretic peptide, blood urea nitrogen:creatinine ratio, and the urinary fractional excretion of sodium. Ten dogs with chronic kidney disease (CKD) with matching degree of azotemia were recruited as controls. The activity of the cRAAS was low in 10 dogs, normal in 3 dogs, high in 1 dog and equivocal (high renin—low aldosterone) in 1 dog with GD. These dogs had a lower cRAAS activity than dogs with CKD (p = 0.01). The clinical evaluation showed 8 hypovolemic and 7 non-hypovolemic dogs; 3 dehydrated, 9 euhydrated and 3 overhydrated dogs. The cRAAS activity was not different between hypovolemic and non-hypovolemic dogs. The down-regulated cRAAS without obvious association with the clinical volume status of these dogs with GD, suggests different mechanisms of fluid volume dysregulation in dogs with GD than previously assumed. This finding however should be confirmed in a focused larger scale study, as it may influence the use of cRAAS blockers as part of the standard therapy of GD in dogs.

## Introduction

Glomerular diseases (GD) are common disorders of renal filtration resulting from immune-mediated, infectious, metabolic, toxic, or genetic diseases. They are considered a leading cause of chronic kidney disease (CKD) in dogs [1, 2]. The altered permselectivity causes proteinuria, protein malnutrition, edema, dyslipidemia, systemic hypertension, thromboembolic events, and renal interstitial fibrosis [3, 4]. The exact pathomechanism of fluid retention in GD however is not conclusively understood. The underfill theory centers on hypoalbuminemia, decreased serum oncotic pressure, extravasation of plasma water, hypovolemia, and activation of the circulating renin-angiotensin-aldosterone-system (cRAAS). Activated cRAAS and enhanced tubular sodium reabsorption improve the intravascular volume deficit at the expense of a further decrease in oncotic pressure and a higher propensity of effusion [5–7]. The overfill theory in contrast focuses inappropriate sodium and water retention, hypertension, and extravascular effusion due to epithelial sodium channels, activated by filtered plasmin [8, 9]. A third theory postulates increased vascular permeability caused by Th2-associated reactions [10]. These differing views complicate the therapeutic approach of the animal with GD and a dysregulated fluid balance. Only anecdotal information is available concerning the fluid status of dogs with GD and evidence on the criteria used for its evaluation are lacking. The published therapeutic guidelines for dogs with GD suggest using the response to fluid or diuretic therapy to guide this assessment in dogs [1]. Understanding the underlying pathomechanisms might help with treatment decisions regarding fluid therapy and RAAS blockade.

Dogs with GD presented to our nephrology service anecdotally have been diagnosed with a low aldosterone concentration and hypovolemia, suggesting a down-regulated cRAAS either leading to hypovolemia or independent of the volume status. The aim of this prospective study was therefore to evaluate the cRAAS activity and its potential relationship with the clinical volume status in dogs with GD. A small number of dogs was chosen to confirm this clinical observation.

## Material and methods

### Animals and diseases

Client-owned dogs with naturally occurring GD presented to the Small Animal Clinic of the Vetsuisse Faculty University of Bern (February 2016—October 2018) were included prospectively. Ethical approval was obtained for all procedures (BE143/16). Glomerular disease was defined by the acute or chronic presence of renal proteinuria, a urinary protein:creatinine ratio (UPC) >2 and a pattern of predominant glomerular proteinuria on SDS-PAGE urinary protein electrophoresis. Glomerular pattern was defined as >80% high and intermediate molecular weight proteins, meaning proteins >40 kDa [11]. Histopathologic evaluation of renal biopsies was not required. Dogs with non-proteinuric chronic kidney disease (CKD) were chosen as positive controls for the measurement of the RAAS activity (sample collection, storing or measurement). These dogs were selected to match the azotemia (same proportion of dogs for each class of azotemia corresponding to the IRIS stages) of dogs with GD to evaluate differences specifically related to the proteinuria. They were selected from the pool of known stable patients presented for regular rechecks of their CKD. The definition of CKD was based on history (known kidney disease for ≥3 months with stable creatinine, stable UPC and stable albumin values), laboratory (creatinine ≥1.33 mg/dl or SDMA ≥15 μg/dl, and urine specific gravity (USG) <1.025), and ultrasound abnormalities consistent with CKD (small kidney size, infarcts, or abnormal renal echotexture) [12]. In none of the dogs the blood work or urinalysis have been suspicious of a glomerular damage on top of their chronic kidney disease. Dogs with a UPC ≥2 were excluded from the CKD group. In 2 dogs with CKD we were able to perform a urine protein electrophoresis, showing a tubular pattern of proteinuria. Further exclusion criteria for all dogs included a body weight <8 kg, clinically relevant cardiac disease susceptible to affect the RAAS, and pretreatment with steroids (14 days), fluids (7 days) or diuretics (7 days). The dogs’ diet was not standardized and RAAS blockade was allowed for dogs treated ≥30 days prior to their inclusion because both treatments belong to the standard therapy of GD and CKD.

### Diagnostic evaluation

Clinical assessment and blood and urine sampling for all analysis were collected at presentation before any therapy was started.

Physical examination including a 9-point body condition score, body weight, and blood pressure measurement was performed in all dogs. Blood pressure was measured and classified according to the ACVIM Consensus recommendations [12]. A CBC, chemistry panel, complete urinalysis, UPC and SDS-AGE urine protein electrophoresis were performed for all dogs. Urine was submitted for culture in dogs with an active urine sediment or clinical evidence of urinary tract inflammation.

Plasma renin activity and serum aldosterone concentrations were measured in all dogs at time of presentation immediately after the blood pressure was measured and the clinical examination was done. Pre-chilled EDTA tubes and pre chilled serum tubes were used and blood was drawn from the cephalic vein. Samples were centrifuged (4°C) immediately in the pre-chilled tubes. EDTA plasma (PRA) and serum (aldosterone) were stored at -80°C (max. 12 and 3 months, respectively) and shipped on dry ice for batched analyses (NationWide Specialist Laboratories, Cambridge, UK). There, PRA was measured by using an Angiotensin 1 ELISA method, following a 90 minute generation step at 4°C and 37°C and serum aldosterone by using a competitive radioimmunoassay labelled with Iodine 125. The following PRA—aldosterone patterns were differentiated: downregulated cRAAS (low—low, low—normal, normal—low); upregulated cRAAS (normal—high, high—normal, high—high); normal cRAAS (normal—normal); or equivocal (low—high, high—low).

Clinical assessment of the vascular volume and hydration status was performed as described in Table 1 by the same 2 clinicians (1 DACVIM; 1 resident ACVIM) for all dogs. Systemic blood pressure was evaluated separately as it can be altered by renal diseases independently of the vascular volume. Systemic blood pressure was measured 3–5 times in lateral recumbency using an oscillometric technique. If values differed more than 20% from each other, the Doppler method was used to confirm the result [13]. The mucous membranes moisture was not included in the assessment of hydration because of the uremic xerostomia that can cause the mucous membranes to be dry even in overhydrated dogs [14].

**Table 1:** Criteria used for the clinical assessment of the vascular volume and hydration status.

**Vascular volume status**	Signs suggestive of			
	hypovolemia		euvolemia	
Heart rate	≥160 bpm		<160 bpm	
Pulse Quality	Weak		Normal or strong	
Capillary refill time	≥2 sec		<2 sec	
Acral temperature	Cold		Normal	
Dogs with ≥1 sign suggestive of hypovolemia were classified as hypovolemic				
Dogs with all variables suggestive of euvolemia were classified as non-hypovolemic				
**Hydration status**	Signs suggestive of			
	dehydration	euhydration		overhydration
Skin turgor	Reduced	Normal		Gelatinous
Eyes	Sunken	Normal		
Peripheral edema		Absent		Present
Effusion (pleural, abdominal)		Absent		Present
Dogs with ≥1 sign suggestive of dehydration were classified as dehydrated				
Dogs with ≥1 sign suggestive of overhydration were classified as overhydrated				
Dogs with no sign of dehydration and no sign of overhydration were classified as euhydrated				

Abbreviations: bpm, beats per minute.

All dogs underwent an echocardiographic evaluation performed by a cardiologist (DECVIM-CA) to exclude relevant cardiac diseases and to determine the following volume-related variables: left atrium: aorta ratio (LA:Ao), left ventricular diameter in diastole (LVDd) and end-diastolic volume index (EDVI). The dogs were placed awake in right lateral recumbency and every variable was measured 3–5 times. Previously published reference values were used for LA:Ao [15] and for LVDd [16]. The EDVI was calculated by the modified Teichholz formula, using LVDd and body surface area (BSA, m^2^): EDVI = [7/(2.4+LVDd)] x LVDd^3^ /BSA [17]. In the absence of published reference values for EDVI calculated with this formula, this variable was only used to compare groups but not to classify the dogs as function of their vascular volume. The Simpson method for EDVI measurement has been validated with reference values for dogs, but it was considered too complex for general clinical practice [17].

Additional laboratory variables reflecting the vascular volume included BUN:creatinine ratio, fractional excretion of sodium in the urine (FE_Na_) and N-terminal pro B-type natriuretic peptide (NT-proBNP, Nation Wide Specialist Laboratories, Cambridge, UK).

### Sample size and statistical analyses

Lacking previous results for sample size estimation, we elected to characterize a small group of 15 dogs with GD not receiving a therapy influencing their volume status. A control group of 10 dogs with CKD and matching azotemia was selected to serve as positive controls of cRAAS activation and to differentiate results specifically associated with the proteinuria.

Statistical analysis was mainly descriptive and statistical significance, set as P<0.05, was only assessed for the comparison of groups with >5 cases. Categorical variables are reported as absolute numbers and proportions (%) and they were compared between groups using a Chi-square test. As most variables were not normally distributed (Shapiro-Wilk test), continuous variables are presented as median and interquartile range (IQR), and comparisons between groups were performed with a Mann-Whitney U test. A regression analysis was used to assess the correlation between FE_Na_ and PRA or aldosterone. Statistical analyses were performed with the statistical package NCSS (NCSS 9 Statistical Software, 2013. NCSS, LLC. Kaysville, Utah, USA).

## Results

### Dogs and diseases

Fifty-eight dogs with GD were screened for inclusion. Nine dogs were excluded because the owners elected not to participate, 11 dogs because of the body weight limit, 5 dogs because of cardiac disease, and 18 dogs because of pretreatment with fluids (n = 13), benazepril (n = 2), or steroids (n = 3). The remaining 15 dogs were enrolled, including 7 stable outpatients and 8 dogs requiring hospitalisation. Thirteen dogs with stable CKD and matching azotemia were considered for inclusion. Ten dogs were included after the exclusion of 3 dogs because of cardiac disease.

The GD group included 5 French bulldogs, 2 Border Collies, 2 mixed breed dogs, and one dog each of 6 different breeds. The group consisted of 7 females (6 spayed) and 8 males (4 castrated), with a median age of 5.5 years (range, 2–11). The CKD group included one dog each of 9 different breeds and one mixed breed dog. It consisted of 6 females (4 spayed) and 4 intact males, with a median age of 4.0 years (range, 0.8–11).

Of the 15 dogs with GD, 12 had evidence of a possible underlying infectious cause: leishmaniasis (6), *Borrelia burgdoferi* seropositivity (n = 5, IDEXX Snap 4Dx PlusTest), and *Anaplasma* spp. seropositivity (n = 1, IDEXX Snap 4Dx PlusTest). Additionally, 1 dog was diagnosed with acute pancreatitis. Renal histopathology was not available for any of the dogs, because of their advanced azotemia and for financial and safety concerns. Of the 10 dogs with CKD, 2 had residual renal impairment following acute kidney injury, 2 had a suspicion of renal dysplasia, 2 were diagnosed with chronic pyelonephritis (inactive at inclusion), 1 with a chronic obstructive uropathy, 1 with multiple renal cysts, and 1 with a previous history of ehrlichiosis. Two of 15 dogs with GD and 3/10 dogs with CKD were pretreated with RAAS blockade: benazepril (0.25–0.9 mg/kg/d for >90 days; n = 4) or losartan (4 mg/kg/d for 40 days; n = 1). Salt restricted renal diet was strictly given to 1 dog with GD and 4 dogs with CKD.

### Clinical and laboratory assessment

Relevant clinical and laboratory variables for both groups are summarized in Tables 2–4. Thirteen of 15 dogs with GD and 8/10 dogs with CKD were azotemic, with no significant difference in the degree of azotemia between the groups. Dogs with GD had a higher systolic blood pressure, lower albumin, higher potassium, higher phosphorus, and higher UPC than dogs with CKD.

**Table 2:** General laboratory variables of 15 dogs with glomerular disease (GD) and 10 dogs with chronic kidney disease (CKD) included in the study.

Variables	GD	CKD	P-value
[reference interval]	(n = 15)	(n = 10)	
**Urea** (mmol/l)	**36.9** (22.2–53.8)	**16.2** (9.3–35.8)	0.18
[3.3–10.8]			
**Creatinine** (μmol/l)	**332** (202–503)	**261** (142–535)	0.64
[52–117]			
**Albumin** (g/l)	**24.2** (17.5–26.8)	**32.7** (30.2–33.4)	<0.001*
[30.0–41.0]			
**Sodium** (mmol/l)	**148** (146–150)	**146** (145–147)	0.17
[142–154]			
**Potassium** (mmol/l)	**4.77** (4.25–5.38)	**4.24** (4.01–4.44)	0.04*
[3.95–5.4]			
**Phosphorus** (mmol/l)	**4.01** (2.04–5.07)	**1.81** (0.87–2.39)	0.01*
[0.91–1.9]			
**SDMA** (mg/dl)	**41.5** (24.5–56.5)	**26.5** (17.8–47.0)	0.13
[<15]			
**UPC**	**10.4** (5.4–17.2)	**0.6** (0.3–1.2)	<0.001*
[<0.2]			

Data are presented as median (IQR). P-values are indicated for the comparison between the groups (Mann-Whitney U test

*, statistically significant with P <0.05).

**Fig 1:**
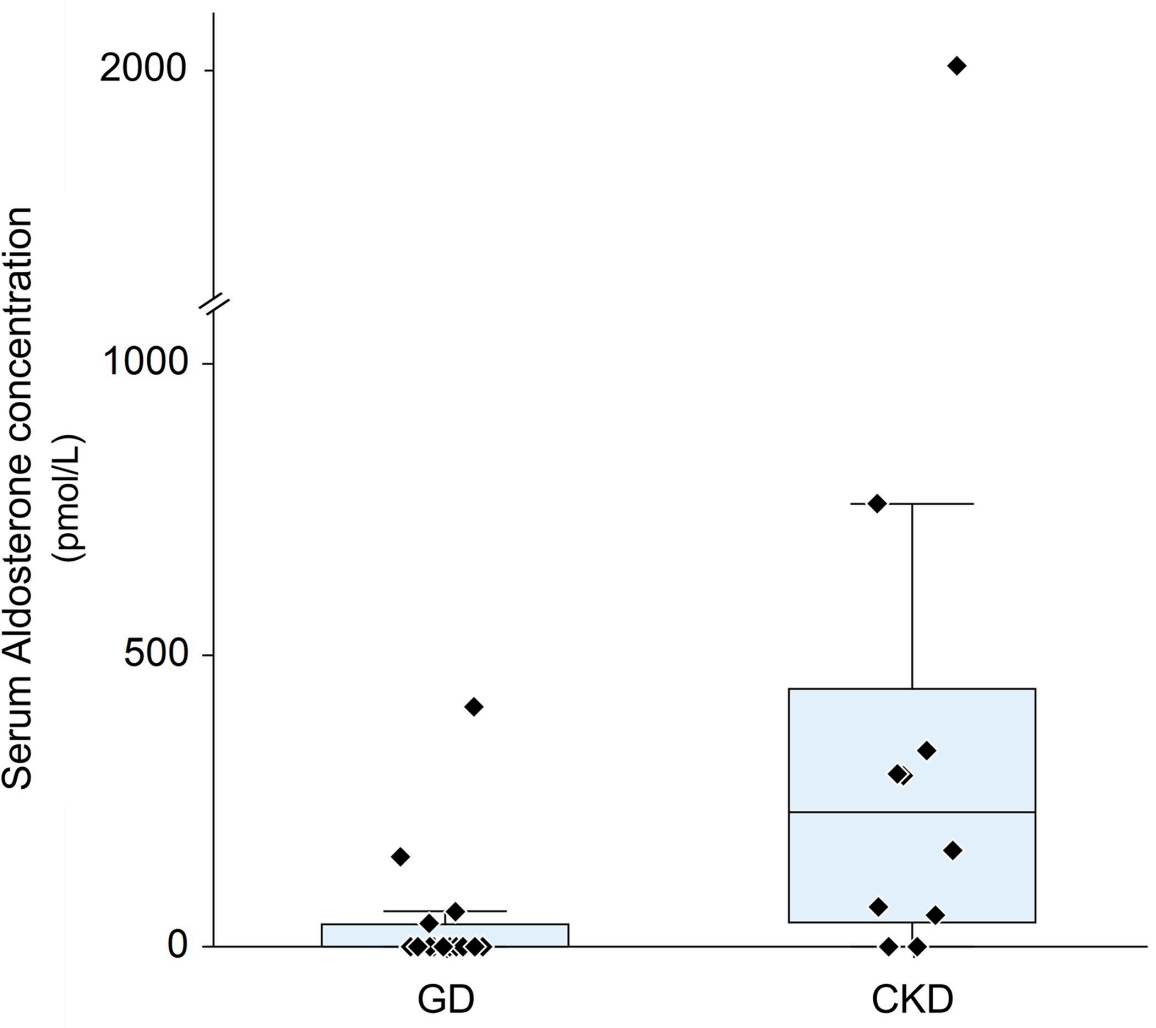
Serum aldosterone concentration. GD = Glomerular disease, CKD = chronic kidney disease. Each diamond represents 1 dog.

**Fig 2:**
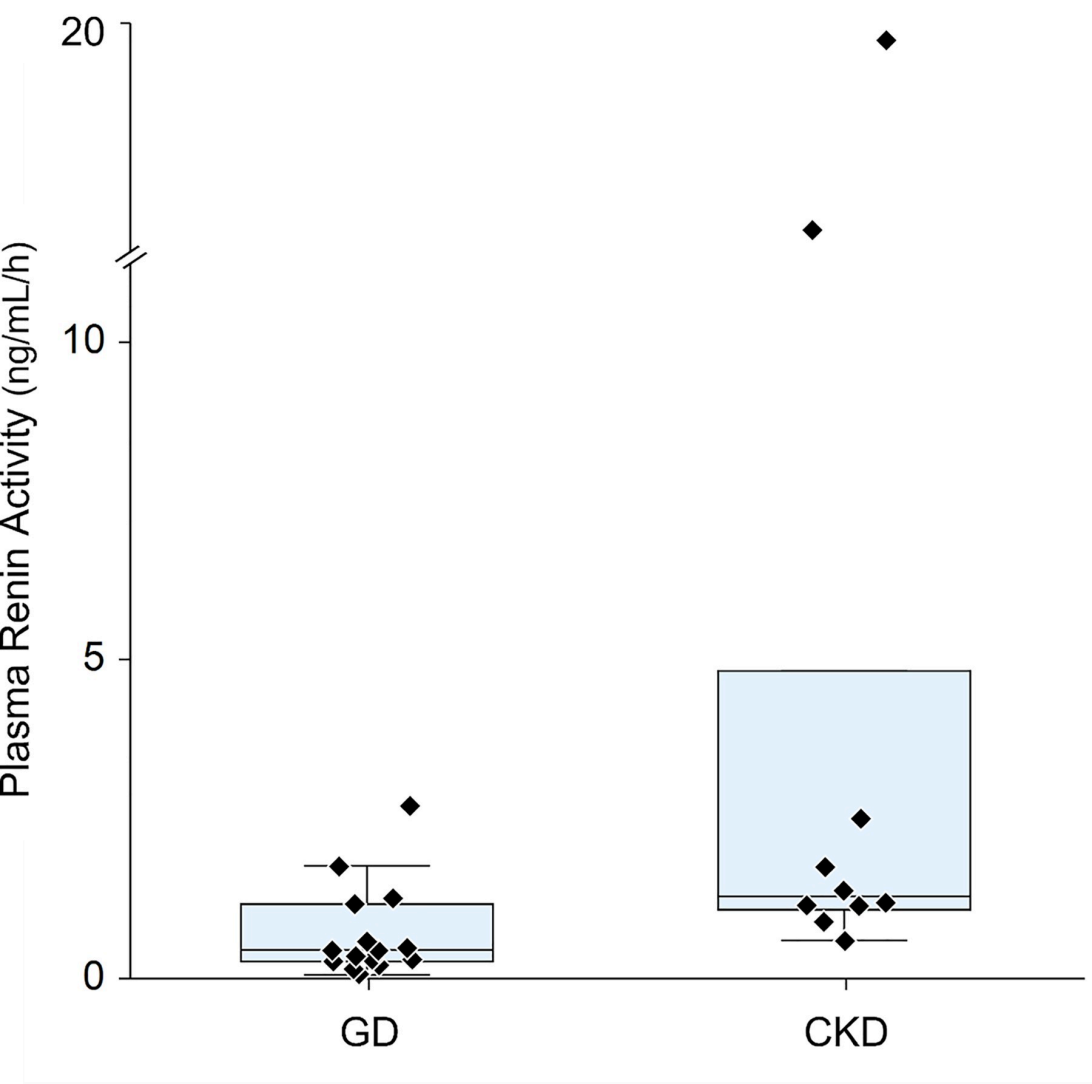
Plasma renin activity. GD = Glomerular disease, CKD = chronic kidney disease. Each diamond represents 1 dog.

Plasma renin activity and serum aldosterone concentration were lower in dogs with GD than in dogs with CKD (p = 0.01 for both; Figs 1 and 2).

**Table 3:** Clinical, echocardiographic, laboratory, and neuroendocrine variables putatively associated with the fluid status in dogs with glomerular disease (GD) or chronic kidney disease (CKD).

	Variables	GD	CKD	P-value
	[reference interval]	(n = 15)	(n = 10)	
**Clinical evaluation**	**Systolic BP** (mmHg)	**170** (156–185)	**146** (137–167)	0.02*
	Normotension	0 (0%)	3 (30%)	
	Pre-hypertension	4 (27%)	4 (40%)	
	Hypertension	6 (40%)	2 (20%)	
	Severe hypertension	5 (33%)	1 (10%)	
**Echocardiography**	**LA:Ao ratio**	**1.13** (1.00–1.18)	**1.17** (1.04–1.25)	0.57
	[0.80–1.60]			
	**LVDd** (cm)	**3.42** (2.75–3.70)	**4.03** (3.45–4.72)	0.07
	**EDVI** (ml/m2)	**67.6** (59.6–90.5)	**78.8** (65.6–104.5)	0.26
**Other variables**	**BUN:creatinine ratio**	**22.8** (13.1–35.3)	**17.4** (13.9–22.8)	0.20
	**NT-proBNP** (pmol/l)	**2459** (329–6751)	**3050** (1716–4371)	0.96
	[800–900]			
	**FE**_**Na**_ (%)	**1.01** (0.33–3.93)	**0.71** (0.49–1.99)	0.63
**RAAS activity**	**Renin activity** (ng/ml/h)	**0.44** (0.27–1.17)	**1.29** (1.08–5.25)	0.01*
	[0.22–2.40]			
	**Aldosterone** (pmol/l)	**0** (0–40)	**229** (41–442)	0.01*
	[0–393]			

Data are presented as median (IQR). P-values are indicated for the comparison between the groups (Mann-Whitney U test

*, significance at P <0.05).

Abbreviations: BUN, blood urea nitrogen; EDVI, end-diastolic volume index; FE_Na_, fractional excretion of sodium; LA:Ao, left atrium:aorta ratio; LVDd, left ventricular volume in diastole; NT-proBNP, N-terminal pro-B-type natriuretic peptide.

**Table 4:** Laboratory, echocardiographic, and neuroendocrine variables putatively associated with the vascular volume status of 15 dogs with GD evaluated clinically as hypovolemic or non-hypovolemic.

Variables	Hypovolemic	Non-hypovolemic	P-value
	(n = 8)	(n = 7)	
**Albumin** (g/l) [30.0–41.0]	**23.8** (15.1–24.9)	**25.7** (19.3–26.8)	0.26
**UPC**	**10.8** (5.6–17.4)	**8.7** (5.3–13)	0.88
**LA:Ao ratio**	**1.09** (0.99–1.18)	**1.13** (1.1–1.18)	0.81
**LVDd** (cm)	**3.51** (3.34–4.69)	**5.09** (4.86–8.68)	0.06
**EDVI** (ml/m2)	**61.9** (58.9–70.3)	**69.5** (67.1–100.9)	0.09
**BUN:creatinine ratio** (mg/dl:mg/dl)	**24.1** (22.4–38.3)	**20.9** (12.6–24.7)	0.4
**NT-proBNP** (pmol/l) [800–900]	**1894** (270.8–7563)	**3510** (1309–5853)	0.94
**Renin activity** (ng/ml/h) [0.22–2.40]	**0.40** (0.26–0.68)	**0.44** (0.3–0.88)	0.85
**Aldosterone** (pmol/l) [0–393]	**0** (0–10)	**0** (0–77)	0.24
**FE Na** (%)	**0.41** (0.28–2.56)	**2.1** (1.16–2.95)	0.27

Data are presented as median (IQR). P-values are indicated for the comparison between the groups (Mann-Whitney U test

*, significance at P <0.05).

Abbreviations: BUN, blood urea nitrogen; EDVI, end-diastolic volume index; FE_Na_, fractional excretion of sodium; LA:Ao, left atrium:Aorta ratio; LVDd, left ventricular volume in diastole; NT-proBNP, N-terminal pro-B-type natriuretic peptide; UPC, urine protein creatinine ratio.

Plasma renin activity was low in 3 dogs, in the reference range in 11 and elevated in 1 dog with GD. Serum aldosterone concentration was undetectably low in 11 dogs, in the reference range in 3 dogs and elevated in 1 dog with GD. Activation patterns were low—low (3), normal—low (7), normal—normal (3), high—low (1) and normal—high (1). Therefore, cRAAS activity was interpreted as downregulated for 10 dogs, normal for 3 dogs, upregulated in 1 dog and equivocal in 1 dog.

Pretreatment with RAAS blockade in 5 dogs resulted in different RAAS patterns: downregulated or normal in each 1 dog with GD; normal in 2 dogs and upregulated in 1 dog with CKD. No dog treated with blockers of the RAAS showed the expected PRA high—aldosterone low-normal pattern. As pretreatment with RAAS blockade could still have had an influence on PRA and aldosterone concentration, we performed the statistics again, excluding those dogs on RAAS inhibitors. Both, PRA and Aldosterone concentrations, remained statistically significant different between dogs with GD and dogs with CKD (PRA: p = 0.029 and Aldosterone: p = 0.028).

The echocardiographic variables provided little evidence of abnormal vascular volume. They were within normal limits in most dogs of both groups and not different between groups (Table 3): LA:Ao was normal in all 25 dogs; LVDd in 14/15 dogs with GD (93%) and 8/10 dogs with CKD (80%). The additional laboratory variables were not different between the groups (Table 3). Interestingly, NT-proBNP was elevated in 18/25 dogs without echocardiographic evidence of cardiac disease or volume overload. The FE_Na_ was neither correlated with the PRA (r^2^ = 0.01, p = 0.70) nor with the serum aldosterone concentration (r^2^ = 0.01, p = 0.67).

Two dogs with GD (13%) were clinically tachycardic, 4 (27%) had weak pulses, 3 (20%) a prolonged CRT, and 4 (27%) cold extremities. Consequently, 8 dogs (53%) were classified as clinically hypovolemic and 7 (47%) non-hypovolemic. Three dogs had clinical signs of dehydration with a decreased skin turgor. Three dogs showed signs of overhydration (20%): peripheral edema (n = 2), gelatinous skin (n = 2), and ascites (n = 1). Three dogs (20%) were therefore classified as dehydrated, 3 (20%) overhydrated and 9 (60%) euhydrated. Hydration and vascular volume status paralleled each other in 8 dogs (53%) and were discordant in 7 dogs (3 hypovolemic euhydrated dogs, 3 hypovolemic overhydrated dogs and 1 non-hypovolemic dehydrated dog). All dogs with CKD were considered non-hypovolemic and euhydrated. Hypovolemic dogs did not differ in any echocardiographic or laboratory variable from non-hypovolemic dogs with GD (Table 4). In particular the renin activity and the aldosterone concentration were not different between these two groups of dogs.

## Discussion

This prospective study showed that dogs with GD can have various patterns of cRAAS activity, not related to their volemia. The cRAAS activity was generally lower in these proteinuric dogs with GD than in non-proteinuric dogs with CKD. In addition, dogs with GD displayed variable alterations of their fluid volume status, including overhydration, dehydration and hypovolemia. However, the laboratory and echocardiographic variables supposed to reflect volemia showed weak agreement with the clinical assessment when considered in isolation.

Fluid status alterations were not recognized clinically in dogs with CKD, possibly due to the restrictive inclusion of clinically stable dogs or to the lack of sensitivity of the clinical assessment. Confirming our subjective observations, PRA and serum aldosterone concentration were lower in dogs with GD than with CKD and similar degrees of azotemia. Only one dog with GD showed the expected upregulated cRAAS activity mentioned as the main target of therapy for this disease [1, 18]. The patterns observed indicated that the cRAAS activity as assessed was not consistently reflecting the vascular volume. Whether this is indicating cRAAS-independent mechanisms in the pathogenesis of fluid disturbances in dogs with GD needs to be investigated in a specifically designed study. The higher potassium concentration of dogs with GD compared to CKD could reflect the lower cRAAS activity of this group. Further insights in the activity of the RAAS in dogs with GD should be gained from a larger group of dogs and from a more detailed investigation such as a RAAS fingerprint analysis [19]. The local renal RAAS may not be reflected in the systemic cRAAS as evaluated here [20] and urinary renin and aldosterone or biopsy-based mRNA expression of the RAAS components could bring further insights in these mechanisms [21]. In the meantime, blockade of the RAAS should remain a mainstay of treatment in dogs with GD as it has been shown beneficial by reducing proteinuria [22].

The lack of correlation between FE_Na_ and PRA or serum aldosterone concentration suggests that renal sodium handling is not exclusively regulated by the RAAS in dogs with GD. Other mechanisms may include proteinuria-induced overexpression of tubular cells ENaC, plasmin-activated epithelial sodium channels [8], a progressive loss in the ability to reabsorb sodium or disturbed pressure natriuresis response. These might be mechanisms of RAAS-independent sodium retention, however, they should cause hypervolemia, while hypovolemia was mainly seen in the dogs with GD in this study. Considering the higher cRAAS activity in the dogs with CKD, the latter mechanism seems unlikely. In addition, we can only speculate a role of the type of GD in our dogs, as renal histopathology was not available. It is however a likely oversimplification to consider all GDs uniformly in terms of volume dysregulation.

Different methods have been used to measure total body water and quantitate hydration. The clinically impractical gold standard techniques of bromide or deuteriated water dilution have been supplanted in humans by indirect methods such as bioimpedance analysis and was therefore not performed in our pilot study [23]. Similarly, the urinary potassium/ (potassium + sodium) ratio has been suggested as a surrogate of hypovolemia in nephrotic children to replace the dilution methods with radioactively labeled red blood cells or proteins [24]. To date, the validity of this method has not been evaluated in companion animals.

The echocardiographic variables evaluated in this study did not prove useful or sensitive enough to assess the vascular volume, most dogs being within the described reference ranges. These broad reference intervals may have to be reviewed however, focusing on narrower breed specific ranges. The lack of agreement of echocardiographic variables and clinical variables such as heart rate or systolic blood pressure was reported previously in dogs [25, 26]. Echocardiographic variables might be useful to follow the development of volemia after initation of therapy (fluid or diuretics) but should not be used alone to estimate the fluid status in dogs presently. The NT-proBNP was not different between the tested groups but 18/25 dogs had elevated concentrations without clinically relevant cardiac disease. This suggests a decreased renal clearance of this marker, although previous studies provided controversial results [27, 28].

A FE_Na_ <1% is considered appropriate in dogs or it can indicate an activated RAAS with increased sodium reabsorption. A FE_Na_ >1% is described in dogs with acute kidney injury [29, 30] and in humans with tubular injury, hypervolemia, or hypertension. The marked overlap between hypovolemic and non-hypovolemic dogs and the lack of correlation with the cRAAS activity make FE_Na_ unlikely to be helpful for the assessment of the volume status in this setting. We cannot rule out an effect of RAAS blockade on sodium excretion, as 5/25 dogs were pretreated with RAAS blocking agents.

A BUN:creatinine ratio >20 suggests dehydration in humans, but it is influenced by other parameters such as dietary protein, gastrointestinal hemorrhage, catabolic rate, and muscle mass [31]. The BUN:creatinine ratio was >20 in all clinically hypovolemic dogs except for 1, and 2/3 clinically dehydrated dogs with GD in our study, but it was also elevated in dogs without signs of dehydration or hypovolemia. In addition, species-, diet-, and body conformation-specific reference intervals are needed for this variable to be evaluated further.

There are several limitations to this pilot study, including the small number of dogs evaluated. This number however proved sufficient to confirm our clinical observation and our main study hypothesis of a low cRAAS activity in dogs with GD. The lack of a clinically applicable gold standard or of a validated system for clinical assessment of volemia limits the clinical conclusions concerning the relationship of cRAAS activity and volume status. The use of serial assessments and the evaluation of the response to fluid therapy suggested by the consensus group [1] could not be used as a standard because most dogs were not hospitalized. Treatment and nutrition were not standardized for practical reasons, but all dogs were fasted for at least 6 hours prior to blood sampling. The evaluation of the cRAAS activity was limited to a single assessment of PRA and serum aldosterone and did not include angiotensin II, angiotensin converting enzyme activity, and urinary renin and aldosterone concentrations [32]. Sampling time was not standardized, disregarding the circadian periodicity of the RAAS [32] and as the diet was not standardized an influence of a different sodium intake cannot be ruled out. In addition, dogs were allowed to be pretreated with RAAS blockers and although only 1 dog was seen with the expected pattern (PRA high, Aldosterone low) we cannot rule out that cRAAS activity was influenced by this pretreatment in 5/25 dogs. Another limitation is the lack of histopathological examination to differentiate between types of GD. Different underlying causes and pathomechanisms may affect differently the cRAAS and the fluid regulation. Furthermore, a misclassification bias may exist as an underlying GD was not ruled out in dogs with CKD by renal biopsies. Last, we matched the two groups only based on the degree of azotemia. However, age and gender have been shown to influence cRAAS activity in humans. We can therefore not rule out an influence of these variables on our results, although both groups had similar age and gender repartition.

In summary, cRAAS activity was low in dogs with GD in contrast to dogs with CKD, although hypovolemia was seen in a majority of these dogs. Various patterns of fluid volume alterations were observed in dogs with GD, including hypovolemia, dehydration and overhydration. This suggests that the mechanisms of fluid volume dysregulation may not be explained uniformly for all dogs of this group. Additionally, renal handling of sodium seems to be influenced by mechanisms other than the cRAAS in these dogs with GD.

In conclusion, the low cRAAS activity in dogs with GD should be evaluated further in a larger study as RAAS inhibition is one of the standard therapies of those dogs. This assessment should include a more detailed assessment of the RAAS components to characterize the dominant alterations and thus precise the mechanisms underlying the observed fluid volume dysregulation.

## Supporting information

S1 File Clinical, laboratory and imaging variables of all dogs. (PDF)

S2 File (PDF)
