# Supplementary material for: The circulating renin-angiotensin-aldosterone system is down-regulated in dogs with glomerular diseases compared to other chronic kidney diseases with low-grade proteinuria
Source: PLoS One. 2022 Jan 10;17(1):e0262121. doi: 10.1371/journal.pone.0262121 (PMC8746712; doi:10.1371/journal.pone.0262121)
Supplement: S1 File — (PDF) [file pone.0262121.s001.pdf]

Tabelle 1

Dog	Group	Hopitsalized	Breed	DOB	DOP	Sex
1	GD	Yes	French Bulldog	2/26/12	2/11/16	Female spayed
2	GD	No	Mixed breed	1/1/14	5/19/16	Male castrated
3	GD	No	Labrador Retriever	12/15/05	4/28/16	Female
4	GD	No	Norsk Buhund	12/15/05	5/27/16	Female spayed
5	CKD	No	Doggue Bordeaux	9/30/14	8/8/16	Male
6	GD	No	Cocker Spaniel	9/12/07	11/3/16	Male
7	GD	No	French Bulldog	8/6/08	11/30/16	Male
8	GD	Yes	Mixed breed	9/6/11	1/25/17	Female spayed
9	CKD	No	Beauceron	9/23/06	2/27/17	Male
10	CKD	No	Bernese Mountain dog	7/30/13	2/7/17	Female spayed
11	GD	No	Border Collie	12/5/11	3/3/17	Male castrated
12	GD	Yes	Border Collie	12/5/11	3/7/17	Male castrated
13	CKD	No	American Bulldog	4/21/09	3/23/17	Male
14	CKD	No	Boxer	2/13/16	4/24/17	Female spayed
15	GD	Yes	French Bulldog	6/15/12	11/6/17	Male castrated
16	CKD	No	Flat Coated Retriever	5/4/17	11/17/17	Female spayed
17	GD	Yes	French Bulldog	9/1/13	1/8/18	Female spayed
18	CKD	No	Border Collie	1/24/11	2/9/18	Female spayed
19	GD	No	French Bulldog	5/26/15	2/8/18	Male castrated
20	CKD	No	Mixed breed	1/1/09	2/2/17	Male
21	GD	Yes	Mastiff	5/16/15	3/27/18	Male
22	GD	Yes	Australian Shepherd	2/19/14	4/9/18	Female spayed
23	CKD	No	Rhodesian Ridgeback	7/29/17	8/24/18	Female
24	GD	Yes	Bernese Mountain dog	10/15/14	10/16/18	Female spayed
25	CKD	No	German Shepherd	9/5/17	10/23/18	Female

BCS=Body condition score

CRF=capillary refill time

DOB=Date of birth

DOP=Date of presentation

UPC=Urinary Protein:creatinine ratio

* blood pressure measured by Doppler method

Dog	Disease	BCS (1-9)	Body weight (kg)	Heart rate	Pulse quality	CRF
1	Leishmaniasis	1	7.9	120	Normal	1.5 sec
2	Leishmaniasis	3	11.2	84	Normal	1.5 sec
3	Pancreatitis	4	26.0	80	Normal	1.5 sec
4	Anaplasmosis	4	13.4	180	Weak	1.5 sec
5	Leptospirosis	5	57.4	100	Normal	1.5 sec
6	Leishmaniasis	4	11.4	116	Normal	1.5 sec
7	Unknown	4	13.2	102	Normal	1.5 sec
8	Unkown	4	27.4	140	Weak	1.5 sec
9	Leptospirosis	4	40.0	110	Normal	1.5 sec
10	Circumcaval Ureter	8	57.0	104	Normal	1.5 sec
11	Leishmaniasis	3	19.0	120	Normal	1.5 sec
12	Leishmaniasis	3	18.2	140	Weak	1.5 sec
13	Renal cysts	3	35.9	104	Normal	1.5 sec
14	Renal dysplasia	4	21.1	100	Normal	1.5 sec
15	Borreliosis	3	11.3	160	Normal	2 sec
16	Renal dysplasia	4	14.4	100	Normal	1.5 sec
17	orreliosis, Polycythem	3	9.7	116	Normal	2 sec
18	naplasmosis, Babesios	4	17.4	130	Normal	1.5 sec
19	Borreliosis	1	7.5	148	Normal	2 sec
20	Unknown	4	9.7	100	Normal	1.5 sec
21	Leishmaniasis	4	68.8	76	Normal	1 sec
22	Borreliosis	4	30.9	80	Weak	1.5 sec
23	Chron. Pyelonephritis	4	28.0	150	Normal	1.5 sec
24	Familial GD	5	7.0	100	Normal	1.5 sec
25	Chron. Pyelonephritis	4	37.9	112	Normal	1.5 sec

Dog	Acral temperature	Skin turgor	Eye balls	Peripheral edema	Effusion	Systolic blood pressure (Median)
1	Cold	Reduced	Normal	None	None	156
2	Normal	Normal	Normal	None	None	185
3	Normal	Normal	Normal	None	None	197
4	Normal	Normal	Normal	None	None	163
5	Normal	Normal	Normal	None	None	140 *
6	Normal	Reduced	Normal	None	None	170 *
7	Normal	Normal	Normal	None	None	240 *
8	Normal	Normal	Normal	Yes	None	165
9	Normal	Normal	Normal	None	None	186
10	Normal	Normal	Normal	None	None	166
11	Normal	Normal	Normal	None	None	142
12	Normal	Reduced	Normal	None	None	176
13	Normal	Normal	Normal	None	None	143
14	Normal	Normal	Normal	None	None	148
15	Cold	Normal	Normal	None	None	220 *
16	Normal	Normal	Normal	None	None	141
17	Cold	Gelatinous	Normal	None	None	166
18	Normal	Normal	Normal	None	None	170 *
19	Cold	Normal	Normal	None	None	150 *
20	Normal	Normal	Normal	None	None	110 *
21	Normal	Normal	Normal	None	None	174
22	Normal	Gelatinous	Normal	Yes	Yes	147
23	Normal	Normal	Normal	None	None	153
24	Normal	Normal	Normal	None	None	184
25	Normal	Normal	Normal	None	None	126

Dog	Mean arterial blood pressure (Median)	Diastolic blood pressure (Median)	Volemia	Hydration	Urea	Creatinine
1	128	114	Hypovolemia	Dehydration	32.2	346
2	128	100	Euvolemic	Normohydration	45.4	503
3	162	144	Euvolemic	Normohydration	36.9	438
4	120	99	Hypovolemia	Normohydration	16.0	112
5			Euvolemic	Normohydration	8.2	152
6			Euvolemic	Dehydration	47.0	202
7			Euvolemic	Normohydration	29.7	273
8	124	104	Hypovolemia	Overhydration	12.8	150
9	131	104	Euvolemic	Normohydration	9.1	151
10	116	92	Euvolemic	Normohydration	9.9	113
11	112	98	Euvolemic	Normohydration	25.4	487
12	145	130	Hypovolemia	Dehydration	39.5	745
13	95	72	Euvolemic	Normohydration	62.3	597
14	106	86	Euvolemic	Normohydration	19.6	344
15			Hypovolemia	Normohydration	60.6	598
16	96	73	Euvolemic	Normohydration	66.2	628
17	110	83	Hypovolemia	Overhydration	62.2	303
18			Euvolemic	Normohydration	12.8	178
19			Hypovolemia	Normohydration	63.2	332
20			Euvolemic	Normohydration	5.8	85
21	133	112	Euvolemic	Normohydration	66.0	1334
22	109	89	Hypovolemia	Overhydration	19.0	206
23	98	71	Euvolemic	Normohydration	40.0	498
24	142	121	Euvolemic	Normohydration	5.6	141
25	94	78	Euvolemic	Normohydration	23.2	514

Dog	Albumin	Sodium	Potassium	Phosphorus	SDMA	UPC
1	24.6	147	4.25	5.00	47.0	11.2
2	26.8	156	6.19	4.24	56.0	4.3
3	18.3	151	4.83	2.92	48.0	6.2
4	25.7	146	4.57	1.25	13.0	5.6
5	33.0	144	4.31	1.70	20.0	1.3
6	25.7	143	5.17	4.01	25.0	8.7
7	33.0	146	4.61	1.49	29.0	3.5
8	8.3	149	4.77	2.04	23.0	17.8
9	33.8	146	4.14	0.88	19.0	1.8
10	36.3	144	4.16	1.18	14.0	0.3
11	17.5	150	4.08	2.92	49.0	15.0
12	17.3	143	5.38	5.30	83.0	10.4
13	30.4	150	3.71	1.91	53.0	0.4
14	29.7	147	4.81	2.17	43.0	0.8
15	27.6	150	5.05	5.46	36.0	5.4
16	33.2	145	4.37	5.21	27.0	0.4
17	23.4	137	6.66	5.07	63.0	3.8
18	24.8	145	4.04	0.83	26.0	0.8
19	24.2	153	3.70	7.28	58.0	17.2
20	32.4	146	3.92	0.83	10.0	0.5
21	26.8	146	5.46	4.12	28.0	44.2
22	7.8	149	3.81	3.37	18.0	27.8
23	33.2	146	4.62	2.58	45.0	1.2
24	20.3	148	4.40	1.41	23.0	11.1
25	31.7	148	4.38	2.32	55.0	0.1

Dog	LA:Ao Ratio (Median)	LVDd (Median)	EDVI (Median)	BUN:creatinine ratio (Median)	NT-proBNP	FE Na
1	1.03	2.70	69.4	23.0	<250	77.0
2	1.20	3.46	101.1	22.4	2290	not done
3	0.98	3.70	66.5	20.9	6095	not done
4	1.00	2.98	61.3	35.4	<250	not done
5	1.25	4.82	72.5	13.4	2099	50.0
6	1.17	2.72	55.1	57.6	329	45.0
7	1.13	3.62	100.7	26.9	276	73.0
8	1.14	3.59	59.6	21.1	361	43.0
9	1.11	4.86	95.0	15.0	3818	18.0
10	1.07	4.30	55.3	21.8	565	81.0
11	1.11	3.42	67.6	13.0	5611	21.0
12	0.97	2.97	49.5	13.2	6751	13.0
13	1.26	4.28	74.5	25.9	6571	0.4
14	1.30	2.95	43.8	14.1	4420	0.5
15	0.94	2.75	57.0	25.1	2459	16.0
16	0.92	3.79	102.8	26.1	2819	12.0
17	1.28	3.19	90.5	50.9	>10000	10.0
18	1.20	3.36	69.1	17.9	2491	61.0
19	1.34	2.73	73.0	47.2	>10000	51.0
20	1.24	3.48	111.8	17.0	305	59.0
21	1.08	4.98	69.5	12.3	>10000	101.0
22	1.15	3.75	62.6	22.8	1329	8.0
23	1.15	4.68	109.5	19.9	3281	37.0
24	1.18	5.13	113.3	9.8	3510	97.0
25	0.97	3.61	83.1	11.2	4354	14.0

Dog	Plasma Renin activity	Aldosterone	Pretreatment
1	0.15	< 20	
2	0.44	<20	
3	0.30	<20	
4	0.44	<20	
5	0.89	165	Telmisartan
6	0.30	154	
7	0.07	<20	ACEi
8	1.26	<20	
9	19.46	759	ACEi
10	0.59	<20	
11	0.58	<20	
12	0.21	<20	
13	1.38	54	
14	2.51	293	
15	0.27	40	
16	13.45	2019	
17	0.35	60	ACEi
18	1.75	68	ACEi
19	0.48	<20	
20	1.14	<20	
21	1.76	411	
22	2.71	<20	
23	1.15	336	
24	1.17	<20	
25	1.19	296	
