# Supplementary material for: The circulating renin-angiotensin-aldosterone system is down-regulated in dogs with glomerular diseases compared to other chronic kidney diseases with low-grade proteinuria
Source: PLoS One. 2022 Jan 10;17(1):e0262121. doi: 10.1371/journal.pone.0262121 (PMC8746712; doi:10.1371/journal.pone.0262121)
Supplement: S2 File — (PDF) [file pone.0262121.s002.pdf]

Intravascular fluid volume assessment of dogs with glomerular disease using clinical, laboratory and imaging parameters

Lisa-Maria Grandt, Ariane Schweighauser, Alan Kovacevic, Thierry Francey
Small Animal Internal Medicine, Vetsuisse Faculty University of Bern, Switzerland

NephroBern

UNIVERSITÄT BERN

Introduction:

Dogs with glomerular disease (GD) may develop nephrotic syndrome. The pathogenesis of edema formation still remains unclear with the underfill theory (renin-angiotensin-aldosterone-system (RAAS) activation through low oncotic pressure) and the overfill theory (activation of epithelial sodium channels and inappropriate sodium conservation) being the main hypotheses, making the choice of treatment difficult (1-5). Assessment of the intravascular volume status of dogs with GD is essential for clinical management. Guidelines and evidence on the choice and the use of available criteria to differentiate hypovolemic-underfilled from hypervolemic-overfilled patients are lacking, as emphasized previously (6). A prospective pilot study was therefore designed to evaluate parameters associated with intravascular volume in dogs with GD compared to non-proteinuric chronic kidney disease (CKD).

Material and methods:

Client owned dogs with natural occurring GD and CKD presented to the Small Animal teaching hospital, Vetsuisse Faculty University of Bern, were prospectively included in this pilot study. Glomerular disease was defined as the presence of significant renal proteinuria with a UPC >2 and a pattern of glomerular proteinuria on urinary protein electrophoresis. Chronic kidney disease was diagnosed using history (persistence of renal azotemia), laboratory data, and ultrasound-based imaging. Recruitment of CKD dogs was performed so that their degree of azotemia matches that of dogs with GD.

Dogs were excluded if they weighed less than 8kg, suffered from cardiac disease or were pretreated with steroids (previous 14 days), fluids or diuretics (previous 7 days). Treatment with RAAS blockers was allowed only if dogs were treated already for at least 7 days prior to inclusion.

Clinical assessment was based on 5 parameters to evaluate volemia (table 1). Dogs with a total score of -1 or less points were defined as hypovolemic, dogs with a total score of 0 or +1 points were defined as non-hypovolemic.

Laboratory parameters were evaluated for their assessment of volemia, including BUN:creatinine, fractional excretion of sodium, NT-proBNP, renin activity, and aldosterone.

Echocardiographic evaluation of the volume status was based on LA:Ao and EDVI (Teichholz method), calculated from LVDd (7).

All parameters were compared between GD and CKD, and between clinically hypovolemic and non-hypovolemic dogs.

Parameter	Heart rate	Pulse quality	Capillary refill time	Acral temperature	Systolic blood pressure	Total score
Points (-1, 0, +1)	-1 or 0	-1 or 0	-1 or 0	-1 or 0	-1 to +1	**-5 to +1**

Table 1

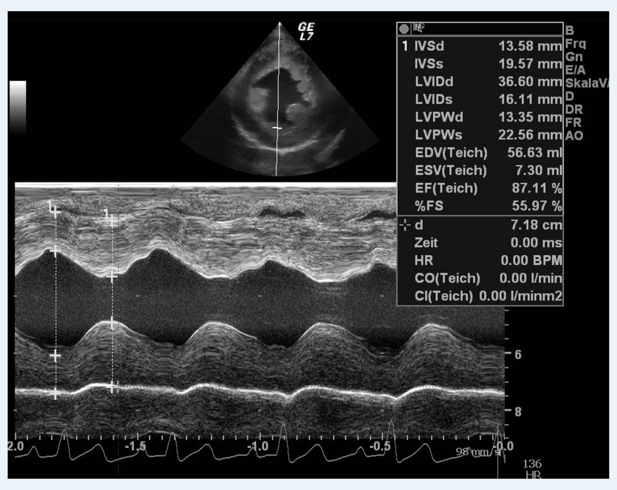

Measurement of LVDd

Results GD vs CKD:

General parameters	GD	CKD	
**SDMA** (mg/dl)	**41.5** (24.5-56.5)	**26.5** (17.8-47.0)	
**Creatinine** (mg/dl)	**3.76** (2.31-5.60)	**2.95** (1.60-6.05)	
**Albumin** (g/l)	**24.2** (17.9-26.3)	**32.7** (30.2-33.4)	**P=0.001**
**UPC**	**10.4** (5.50-16.1)	**0.64** (0.34-1.19)	

Data presented as: Median (IQR)

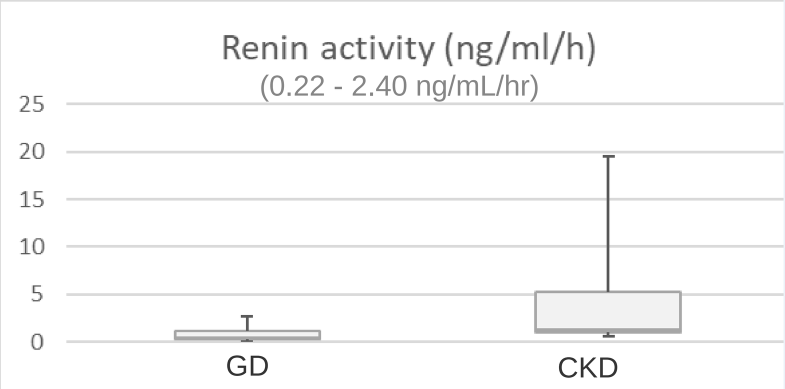

Volume parameters	GD	CKD	
**Clinical hypovolemia**	**6**/15	**0**/10	
**Systolic blood pressure** (mmHg)	**170** (160-184)	**146** (140-163)	**P=0.01**
**Systemic hypertension**	**11**/15	**3**/10	
**LA:Ao**	**1.13** (1.00-1.18)	**1.17** (1.04-1.25)	
**EDVI** (ml/m²)	**67.6** (59.6-90.5)	**78.8** (65.6-104)	
**BUN:creatinine**	**22.8** (13.2-35.4)	**17.5** (14.0 -22.8)	
**FE Na** (%)	**1.01** (0.33-3.93)	**0.71** (0.49-1.99)	
**NT-proBNP** (pmol/l)	**2459** (329-6751)	**3050** (1716-4371)	
**Renin activity** (ng/ml/h)	**0.44** (0.27-1.17)	**1.29** (1.08-5.25)	**P=0.04**
**Aldosterone** (pmol/l)	**0** (0-40)	**165** (27-548)	**P=0.04**

Data presented as: Median (IQR) or as proportion of affected dogs

15 dogs with GD (3 nephrotic) and 10 dogs with CKD were included.

2/15 GD dogs and 3/10 CKD dogs were pretreated with RAAS blockade.

Elevated NT-proBNP was seen without evidence of cardiac disease.

LA:Ao and EDVI were within described limits in 14/15 GD and 8/10 CKD dogs.

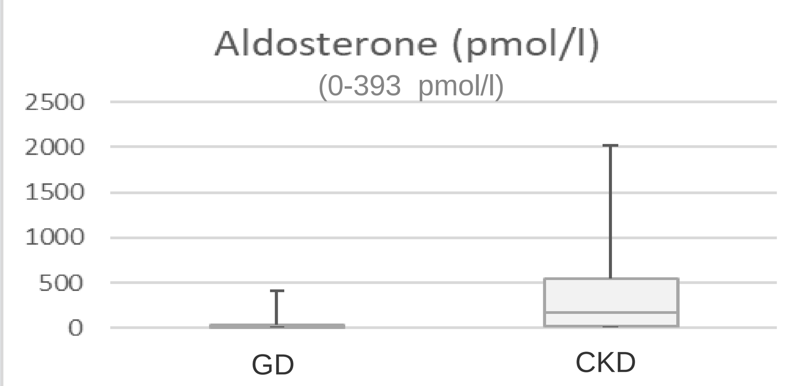

Results hypovolemic vs. non-hypovolemic:

General parameters	Hypovolemic GD	Non-hypovolemic GD
**SDMA** (mg/dl)	**47.0** (36.0-58.0)	**29.0** (25.0-49.0)
**Creatinine** (mg/dl)	**3.59** (2.60-3.87)	**4.95** (2.29-5.69)
**Albumin**	**24.4** (23.6-25.4)	**25.7** (18.3-26.8)
**UPC**	**8.43** (5.44-15.7)	**9.56** (5.76-15.7)

Data presented as: Median (IQR)

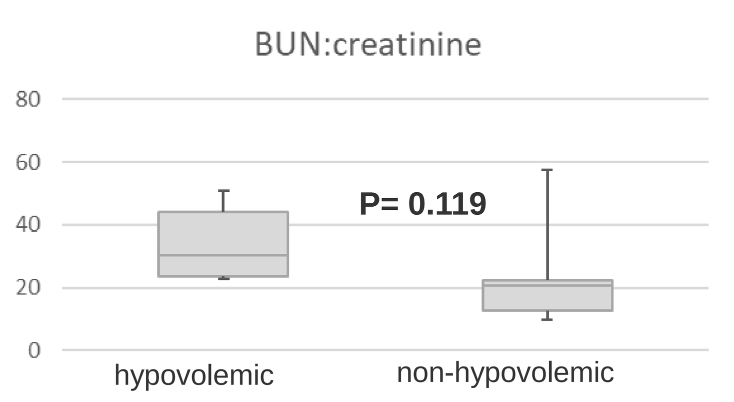

Volume parameters	Hypovolemic GD	Non-hypovolemic GD
**Clinical hypovolemia**	6/15	**9**/15
**Syst. blood pressure** (mmHg)	**160** (152-165)	**176** (170-185)
**LA:Ao**	**1.09** (1.01-1.22)	**1.13** (1.08-1.17)
**EDVI** (ml/m²)	**66.0** (61.6-72.1)	**67.6** (59.6-101)
**BUN:creatinine**	**30.2** (23.6-44.2)	**20.9** (13.0-22.4)
**FE Na** (%)	**0.87** (0.30-4.26)	**1.16** (0.44-2.53)
**NT-proBNP** (pmol/l)	**1894** (332-8115)	**3510** (361-6095)
**Renin activity** (ng/ml/h)	**0.40** (0.29-0.47)	**0.44** (0.30-1.17)
**Aldosterone** (pmol/l)	**0** (0-30.0)	**0** (0-0)

Data presented as: Median (IQR) or as proportion of affected dogs

Discussion/Conclusion:

Despite differences in clinical parameters, dogs with GD had only marginal differences in laboratory and imaging parameters supposed to reflect their intravascular volume status. Volume evaluation of dogs with GD remains difficult and should be based on serial assessment as previously reported (6).

Inactivation of RAAS was an unexpected finding in 13/15 dogs with GD, and would support the overfill theory for edema formation or effusion.

The elevated NT-proBNP could indicate a correlation to the decreased GFR but further investigation is necessary (8).

Elevated BUN:creatinine could indicate hypovolemia but further research in larger study groups is necessary.

References:

(1) Camici M. Molecular pathogenetic mechanisms of nephrotic edema: progress in understanding. BPJ, 2005. Vol. 55 p.215–223

(2) Vaden SL, Pressler BM. Glomerular disease and nephrotic syndrome. In: Bonagura JD, Twedt DC, editors. Kirk's Current Veterinary Therapy XV, 2013. chap.188

(3) Langston C. Managing fluid and electrolyte disorders in renal failure, Vet Clin North Am Small Anim Pract, May 2008 Vol.38:3 p677

(4) Manning Jr RD. Effects of hypoproteinemia on renal hemodynamics, arterial pressure, and fluid volume. Am J Physiol 1987; 252(1 Pt 2): F91–F98.

(5) Svenningsen P, Uhrenholt TR, Palarasah Y, Skjodt K, Jensen BL, Skott O. Prostasin-dependent activation of epithelial Na+ channels by low plasmin concentrations. Am J Physiol Regul Integr Comp Phys- iol. 2009;297(6):R1733-R1741

(6) IRIS Canine GN Study Group Standard Therapy Subgroup, Brown S, chair, Elliott J, Francey T, Polzin D, Vaden SL. Consensus recommendations for standard therapy of glomerular disease in dogs, J Vet Intern Med 2013; 27:S27–S43

(7) Cornell CC, Kittleson MD, Torre PD, Häggström J, Lombard CW, Pedersen HD et al. Allometric scaling of M-Mode cardiac measurements in normal adult dogs. J Vet Intern Med, 2004;18:311-321

(8) Pelander L , Häggström J, Ley CJ, and Ljungvall I. Cardiac troponin I and amino-terminal pro B-type natriuretic peptide in dogs with stable chronic kidney disease. J Vet Intern Med, 2017;31:805–813

Conflict of interest:

We do not declare any conflict of interest.

Universität Bern | Universität Zürich
vetsuisse-fakultät
